# Infectious bursal disease virus induces GSDME-dependent pyroptosis via the mitochondrial caspase-9/3 pathway in DF-1 cells

**DOI:** 10.1186/s13567-026-01815-w

**Published:** 2026-08-12

**Authors:** Liuyang Yuan, Hongchun Yang, Shuangshuang Ma, Aobo Xu, Shufeng Feng, Xiaoran Xuan, Shijun Xu, Songlin Yang, Kairu Wang, Gonghe Li, Hongbin Si, Jianni Huang, Yang Li, Changbo Ou

**Affiliations:** 1 College of Animal Science and Technology, Guangxi University, Nanning, 530004 China; 2 China Animal Health and Epidemiology Center, Qingdao, 266032 Shandong China; 3 Guangxi Zhuang Autonomous Region Engineering Research Center of Veterinary Biologics, Nanning, 530004 China; 4 Guangxi Key Laboratory of Animal Reproduction, Breeding and Disease Control, Nanning, 530004 China

**Keywords:** Infectious bursal disease virus, GSDME, mitochondrial dysfunction, Pyroptosis, VP2, VP5

## Abstract

Infectious bursal disease virus (IBDV) is an important avian pathogen that damages the bursa of Fabricius and continues to threaten the poultry industry. Pyroptosis is an inflammatory form of programmed cell death, but its contribution to IBDV-induced cellular injury remains incompletely defined. Here, using DF-1 cells, we found that IBDV infection was associated with caspase-3 activation, cleavage of gasdermin E (GSDME), generation of the pore-forming N-terminal fragment (GSDME-N), increased lactate dehydrogenase release, plasma membrane damage, and enhanced inflammatory cytokine-associated readouts, including increased interleukin (IL)-1β secretion and elevated IL-18 messenger RNA (mRNA) expression. IBDV infection also induced mitochondrial dysfunction, as evidenced by loss of mitochondrial membrane potential (ΔΨm), increased reactive oxygen species production, and activation of caspase-9, supporting the involvement of a mitochondria-associated caspase-9/3 pathway. Pharmacological inhibition of caspase-9 or caspase-3 reduced pyroptosis-associated readouts, indicating that mitochondrial damage contributes to IBDV-induced lytic cell death under the tested conditions. Expression of viral protein (VP)2 or VP5 alone caused detectable cytotoxicity but did not robustly reproduce GSDME cleavage, suggesting that viral proteins may contribute to upstream cellular stress that is further amplified during infection. Together, these findings support that IBDV engages a mitochondria-associated caspase-9/3–GSDME axis to promote pyroptosis-like lytic cell death in DF-1 cells.

**Supplementary Information:**

The online version contains supplementary material available at 10.1186/s13567-026-01815-w.

## Introduction

Infectious bursal disease virus (IBDV) is a major avian pathogen that primarily targets immature B lymphocytes in the bursa of Fabricius and causes severe immunosuppression in chickens [1–4]. Field studies have shown that variant IBDV infection is associated with bursal atrophy, impaired broiler performance, increased mortality, and economic losses in broiler farms [5]. Moreover, IBDV-induced immunosuppression can weaken vaccine protection, and emerging variant strains can still cause marked bursal damage in immunized flocks, further complicating disease prevention and control [6–8].

The pathological consequences of IBDV infection are closely linked to lymphocyte depletion in the bursa of Fabricius. Apoptosis has long been considered a major mechanism underlying this loss, especially the extensive depletion of B lymphocytes that contributes to immune dysfunction [1–3]. Histopathological studies have demonstrated prominent apoptosis and lymphoid depletion in the bursa after IBDV infection [2, 3], and virulent IBDV infection has been associated with bursal inflammatory injury, cytokine dysregulation, and apoptosis [8]. At the molecular level, IBDV-induced cell death is influenced by both host factors and viral proteins. For example, IBDV infection can enhance microRNA (miR)-16-5p expression in DF-1 cells, thereby promoting apoptosis and viral replication, while host genetic background can shape early immune responses to infection [9].

Several IBDV proteins have been implicated in the regulation of host cell death. VP2, the major capsid protein, has been reported to induce apoptosis [10], whereas the nonstructural protein VP5 may inhibit apoptosis during the early stage of infection [11]. VP3 can also delay premature death of infected cells by inhibiting protein kinase R (PKR)-mediated apoptosis [12]. In addition, the interaction between VP5 and voltage-dependent anion channel 2 (VDAC2) links IBDV infection to mitochondrial regulation of cell death [13]. Together, these findings suggest that IBDV-induced cell death is not restricted to classical apoptosis and may depend on both infection stage and specific viral determinants.

Unlike apoptosis, pyroptosis is a lytic and inflammatory form of programmed cell death characterized by gasdermin-mediated pore formation and plasma membrane rupture [14, 15]. Although gasdermin D is the best-characterized executor of canonical pyroptosis [15], GSDME can also be cleaved by caspase-3, thereby converting apoptosis-associated signaling into a pyroptosis-like outcome with membrane permeabilization [16, 17]. Recent evidence in avian cells indicates that chicken GSDME is an important pore-forming effector during RNA virus infection, and IBDV has been reported to induce chicken GSDME (chGSDME)-dependent lytic cell death in DF-1 cells [18].

Gasdermin-mediated membrane damage may also interact with mitochondrial stress pathways. GSDME pores can permeabilize mitochondria, promote cytochrome c release and further amplify caspase-3 activation, thereby linking mitochondrial dysfunction with lytic cell death execution [19]. However, it remains unclear whether mitochondrial dysfunction contributes to activation of the caspase-9/3–GSDME axis during IBDV infection and whether individual viral proteins participate in this upstream stress response. Therefore, this study investigated whether IBDV induces GSDME-dependent pyroptosis in DF-1 cells through a mitochondria-associated caspase-9/3 pathway and further explored the possible contributions of VP2 and VP5 to this process.

## Materials and methods

### Cells and virus

DF-1 chicken embryo fibroblast cells were maintained in Dulbecco’s modified Eagle’s medium (DMEM; Biosharp, China) supplemented with 10% fetal bovine serum (FBS; PAN-Seratech, Germany), 100 U/mL penicillin, and 100 μg/mL streptomycin at 37 °C in a humidified incubator with 5% CO_2_. The cell-adapted IBDV vaccine strain B87 was preserved in our laboratory at Guangxi University, China. This strain was used as a laboratory-adapted vaccine strain and was not classified as a very virulent field isolate in the present study. Viral titers were determined in DF-1 cells as the 50% tissue culture infectious dose (TCID_50_) using the Reed–Muench method. The viral stock used for infection had a titer of 2.5 × 10^9^ TCID_50_/mL, and virus inocula were prepared according to this TCID_50_ titer to achieve the indicated multiplicity of infection (MOI). For infection, cells were incubated with virus for 2 h to allow adsorption, followed by replacement with maintenance medium containing 2% FBS. Mock-infected control cells were processed in parallel using the same adsorption and medium-replacement procedure without virus.

### Reagents and antibodies

Cell Counting Kit-8 (CCK-8), lactate dehydrogenase (LDH) cytotoxicity kit, Annexin V–fluorescein isothiocyanate (FITC)/propidium Iodide (PI) apoptosis kit, JC-1 mitochondrial membrane potential kit, and caspase-3/-9 activity kits were purchased from Beyotime (China). Lipofectamine 3000, Lipofectamine RNAiMAX, and Opti-MEM were from Thermo Fisher Scientific (USA). MitoSOX Red mitochondrial superoxide indicator was purchased from Beyotime (China). The mitochondrial division inhibitor Mdivi-1 and caspase inhibitors Z-DEVD-FMK (caspase-3) and Z-LEHD-FMK (caspase-9) were from Selleck (China). Unless otherwise indicated, reagents were of analytical grade.

Primary antibodies used for immunoblotting included rabbit anti-GSDME (Abcam, cat. no. ab215191; 1:2000), rabbit anti-FLAG (Beyotime, cat. no. AB3058; 1:5000), and rabbit anti-reduced glyceraldehyde 3-phosphate dehydrogenase (GAPDH) antibody (Proteintech, cat. no. 10494-1-AP; 1:5000). Horseradish peroxidase (HRP)-conjugated anti-rabbit immunoglobulin G (IgG) secondary antibody (Cell Signaling Technology, USA; cat. no. 7074; 1:5000) was used for signal detection. GAPDH served as the loading control for immunoblotting.

### Plasmid transfection

The coding sequences of IBDV VP2 and VP5 were derived from the IBDV B87 strain and cloned into an N-terminal p3 × FLAG-CMV mammalian expression vector to generate N-terminal 3 × FLAG-tagged VP2 and VP5 expression plasmids. The resulting plasmids were designated p3 × FLAG-CMV-IBDV-B87-VP2 and p3 × FLAG-CMV-IBDV-B87-VP5, respectively. In these constructs, the viral protein coding sequences were placed downstream of the CMV promoter and fused in-frame with the N-terminal 3 × FLAG epitope tag. The FLAG tag refers to a short epitope tag used for detection of recombinant protein expression by anti-FLAG immunoblotting.

The VP2 and VP5 inserts were verified by sequencing before use, and the corresponding empty p3 × FLAG-CMV vector was used as the negative control. DF-1 cells were transfected with the indicated plasmids using Lipofectamine 3000 according to the manufacturer’s instructions. Unless otherwise indicated, cells were seeded in six-well plates and transfected at approximately 70–80% confluence. For single-plasmid expression, 2.5 μg plasmid DNA was used per well. For coexpression experiments, equal amounts of VP2 and VP5 plasmids were co-transfected, and the total plasmid amount was kept constant by adding the corresponding empty vector when necessary. Cells were harvested at 24 or 36 h post-transfection for the indicated assays.

### Infection conditions and inhibitor treatments

Cells were infected at an MOI of 5 for immunoblotting and other early assays to minimize rapid cytotoxicity; for cytokine release and other late-stage assays, an MOI of 10 was used unless otherwise stated. For inhibitor experiments, cells were pretreated with inhibitors for 1 h prior to infection or transfection, and the inhibitors were maintained in the medium thereafter unless otherwise indicated. The final concentrations were as follows: Mdivi-1, 20 μM; Z-DEVD-FMK, 20 μM; Z-LEHD-FMK, 20 μM. Equivalent volumes of vehicle (dimethyl sulfoxide (DMSO)) were added to control groups, with the final DMSO concentration kept constant at ≤ 0.1% across all conditions.

### Annexin V-FITC/PI staining

DF-1 cells were seeded in six-well plates at 6 × 10^5^ cells per well and infected with IBDV at the indicated MOI or transfected with the indicated plasmids. For IBDV infection experiments, cells were collected at 6, 24, and 36 h post-infection. For plasmid transfection experiments, cells were collected at 36 h post-transfection. Mock-infected cells or empty vector-transfected cells were used as negative controls. Cells were stained using an Annexin V-FITC/PI apoptosis detection kit according to the manufacturer’s instructions and analyzed using a BD FACSCalibur flow cytometer. Data were processed using FlowJo v10. PI-positive populations were used to evaluate loss of plasma membrane integrity.

### Confocal microscopy

DF-1 cells grown on glass coverslips were infected with IBDV or transfected with the indicated plasmids. Unless otherwise stated, IBDV-infected cells were imaged at 24 h post-infection, and plasmid-transfected cells were imaged at 36 h post-transfection. Plasma membrane morphology was visualized by 3,3′-dioctadecyloxacarbocyanine perchlorate (DiO) staining with 4′,6-diamidino-2-phenylindole (DAPI) counterstaining. Mitochondrial membrane potential was assessed using a JC-1 assay kit, and mitochondrial reactive oxygen species (ROS) was detected using MitoSOX Red according to the manufacturers’ instructions. Mock-infected cells and empty vector-transfected cells were used as negative controls. Fluorescence images were acquired using an Olympus FV5000 confocal microscope under comparable acquisition settings within each experiment.

### siRNA knockdown of GSDME

Short interfering RNAs (siRNAs) targeting chicken GSDME (DFNA5) were designed and synthesized by Sangon Biotech, Shanghai, China. siRNA transfection was performed using Lipofectamine RNAiMAX according to the manufacturer’s instructions. A non-targeting siRNA was used as the negative control (siNC). After siRNA transfection, cells were infected with IBDV or subjected to the indicated treatments according to the experimental design. Cells were harvested at the indicated time points for LDH release, PI uptake, or immunoblotting assays. The siRNA sequences are provided in Additional file 2.

### Western blotting

Cells were lysed in radioimmunoprecipitation assay (RIPA) buffer (50 mM Tris–HCl, pH 8.0; 150 mM NaCl; 1% NP-40; 5 mM ethylenediaminetetraacetic acid (EDTA); 10% glycerol) supplemented with protease inhibitors. Unless otherwise indicated. IBDV-infected cells were collected at 24 h post-infection, and plasmid-transfected cells were collected at 24 or 36 h post-transfection according to the experimental design. Equal amounts of protein were separated by 12% sodium dodecyl sulfate (SDS)-polyacrylamide gel electrophoresis (PAGE) and transferred onto polyvinylidene fluoride (PVDF) membranes. Membranes were blocked with 5% nonfat milk and incubated with the indicated primary antibodies, followed by HRP-conjugated secondary antibodies. Signals were developed using an enhanced chemiluminescence (ECL) substrate. Antibodies against GSDME, FLAG, and GAPDH were used as indicated, and GAPDH served as the loading control.

### LDH release and cell viability

Cytotoxicity and cell viability were assessed using an LDH release kit and a CCK-8 assay kit (Beyotime, China), respectively, according to the manufacturer’s instructions. For IBDV infection experiments, assays were performed at the indicated time points after infection, including 6, 12, 24, 36, and 48 h post-infection. For plasmid transfection and inhibitor experiments, assays were performed at the times specified in the corresponding figure legends. Mock-infected cells, empty vector-transfected cells, and DMSO-treated cells were used as the appropriate negative or vehicle controls. Absorbance was measured at 490 nm for the LDH assay and 450 nm for the CCK-8 assay. All experiments were independently repeated at least three times.

### IL-1β ELISA

Secreted IL-1β in culture supernatants was quantified using a chicken IL-1β enzyme-linked immunosorbent assay (ELISA) kit (Meimian, China; cat. no. MM-3691002) according to the manufacturer’s instructions. Culture supernatants were collected from mock-infected and IBDV-infected cells at 12, 24, 36, and 48 h post-infection. Absorbance was measured at 450 nm using a microplate reader, and IL-1β concentrations were calculated from a standard curve. All experiments were independently repeated at least three times.

### Caspase activity assays

Caspase-3 and caspase-9 activities were measured using colorimetric assay kits (Beyotime, China) with DEVD-pNA and LEHD-pNA as substrates, respectively, according to the manufacturer’s instructions. Cells were collected at 24 h post-infection. Mock-infected cells, empty vector-transfected cells, and DMSO-treated cells were used as the corresponding controls. Absorbance was recorded at 405 nm. All experiments were independently repeated at least three times.

### Quantitative real-time PCR

Total RNA was extracted using TRIzol reagent (Invitrogen, cat. no. 15596018) from mock-infected and IBDV-infected cells at 12, 24, 36, and 48 h post-infection, or from plasmid-transfected cells at the time points specified in the corresponding figure legends. RNA was reverse-transcribed into complementary DNA (cDNA) using a PrimeScript RT kit (Takara, cat. no. RR037A) according to the manufacturer’s instructions. Quantitative polymerase chain reaction (PCR) was performed using SYBR qPCR Mix (Vazyme, cat. no. Q711-02) in a 20-μL reaction volume on a LightCycler 480 system (Roche) with the following cycling conditions: 95 °C for 30 s, followed by 40 cycles of 95 °C for 15 s, 60 °C for 30 s, and 72 °C for 30 s, followed by melting-curve analysis. GAPDH was used as the internal control, and relative gene expression was calculated using the 2^−ΔΔCt^ method. Primer sequences are provided in Additional file 1. Each condition contained *n* = 3 biological replicates, and experiments were independently repeated at least three times.

### Mitochondrial membrane potential (JC-1)

Mitochondrial membrane potential was assessed using a JC-1 assay kit. Cells were incubated with JC-1 working solution for 15 min at 37 °C according to the manufacturer’s instructions and then imaged by fluorescence microscopy or analyzed by flow cytometry as indicated. Unless otherwise stated, IBDV-infected cells were examined at 24 h post-infection, and plasmid-transfected cells were examined at 36 h post-transfection. A decrease in the red/green fluorescence ratio was interpreted as a reduction in mitochondrial membrane potential.

### Propidium iodide (PI) staining

Cells subjected to the indicated treatments were incubated with propidium iodide (PI) solution for 10 min at 37 °C and examined under a fluorescence microscope. PI staining was performed at 24 h post-infection for IBDV infection assays or at 36 h post-transfection for plasmid expression assays. PI-positive cells were considered to have compromised plasma membrane integrity.

### Statistical analysis

Data are presented as mean ± standard deviation (SD). For LDH release, CCK-8 assays, JC-1 red/green fluorescence ratios, and related quantitative assays, each condition contained *n* = 6 biological replicates. For qPCR, caspase-3/9 activity assays, and IL-1β ELISA, each condition contained *n* = 3 biological replicates. All experiments were independently repeated at least three times. Statistical analyses were performed using GraphPad Prism 9.0 with Student’s *t*-test for comparisons between two groups or one-way analysis of variance (ANOVA) for multiple-group comparisons, followed by appropriate post hoc tests. A two-sided *P* < 0.05 was considered statistically significant.

## Results

### IBDV infection reduces DF-1 cell viability and compromises plasma membrane integrity

**Figure 1:**
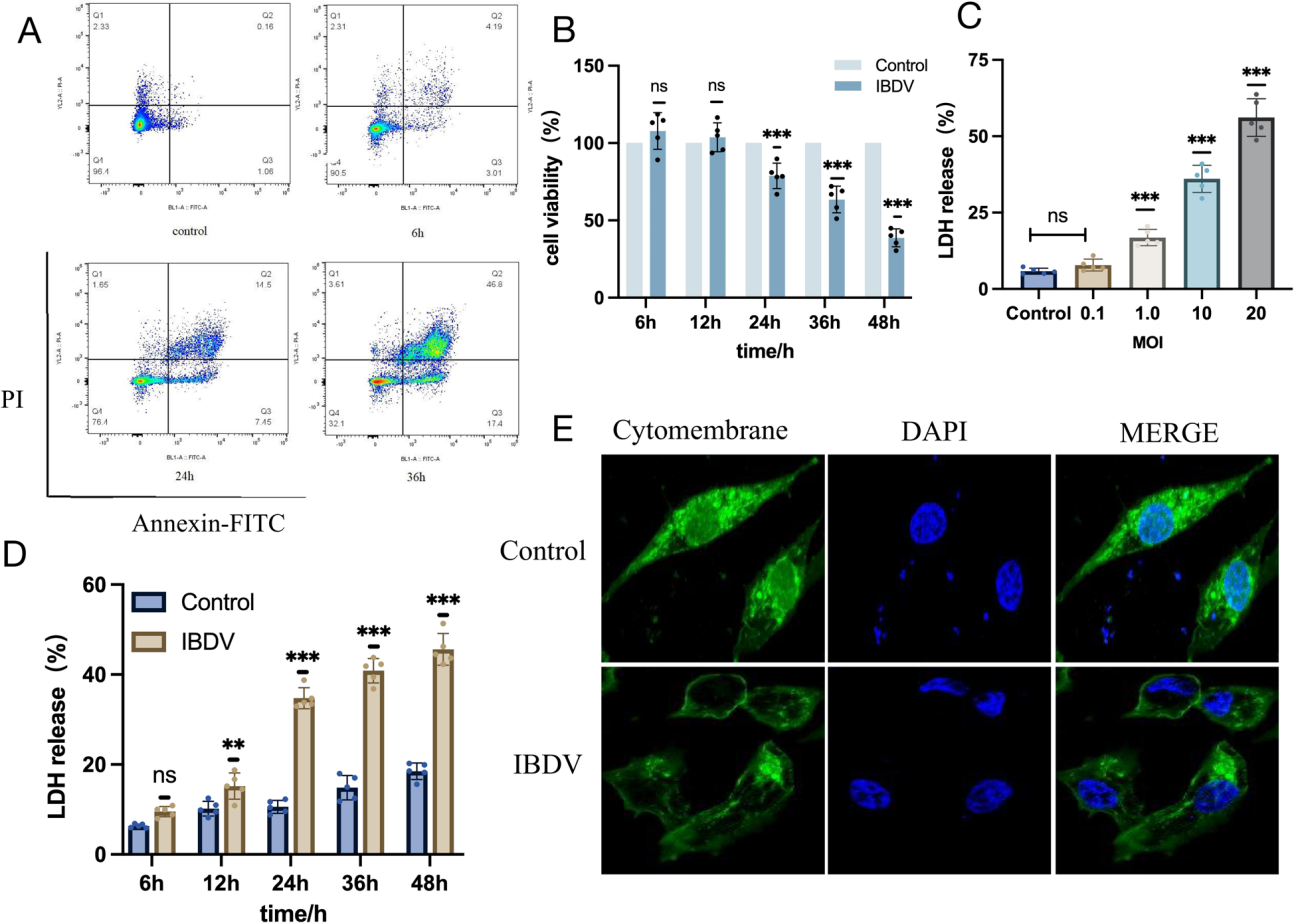
**IBDV infection reduces DF-1 cell viability and compromises plasma membrane integrity.** **A** Annexin V/PI flow cytometry in mock-infected and IBDV-infected DF-1 cells at 6, 24, and 36 h post-infection (MOI = 10). **B** CCK-8 cell viability in mock-infected and IBDV-infected DF-1 cells at 6, 12, 24, 36, and 48 h post-infection (MOI = 10). **C** LDH release in DF-1 cells infected with IBDV at the indicated MOIs. **D** Time-course analysis of LDH release in mock-infected and IBDV-infected cells at 6, 12, 24, 36, and 48 h post-infection (MOI = 10). **E** Confocal microscopy of DiO-labeled plasma membrane (green) and nuclei (DAPI, blue) in mock-infected and IBDV-infected cells at 24 h post-infection (MOI = 10). Data in **B**–**D** are means ± SD (*n* = 6 biological replicates). Flow plots and microscopy images are representative of three independent experiments. ns, not significant; **P < 0.01; ***P < 0.001. Statistics: two-way ANOVA with Šidák’s multiple-comparisons test (**B** and **D**) and one-way ANOVA with Dunnett’s multiple-comparisons test (**C**).

As shown in Figure 1A, Annexin V/PI flow cytometry revealed a time-dependent increase in PI-positive cells following IBDV infection (MOI = 10) compared with mock-infected controls, indicating progressive plasma membrane permeabilization. Consistently, CCK-8 assays showed no significant change at 6–12 h post-infection but a significant decrease in cell viability from 24 h onward (Figure 1B). In parallel, LDH release increased in a dose-dependent manner across the indicated multiplicities of infection (MOIs) (Figure 1C) and rose over the course of infection at MOI = 10 (Figure 1D), consistent with loss of membrane integrity and leakage of cytosolic contents. Confocal imaging further showed marked disruption of membrane morphology in infected cells compared with controls (Figure 1E). Collectively, these data indicate that IBDV infection impairs DF-1 cell viability and causes pronounced membrane damage accompanied by LDH release, consistent with a lytic cell death phenotype.

### IBDV infection induces GSDME-dependent pyroptosis accompanied by inflammatory cytokine responses

**Figure 2:**
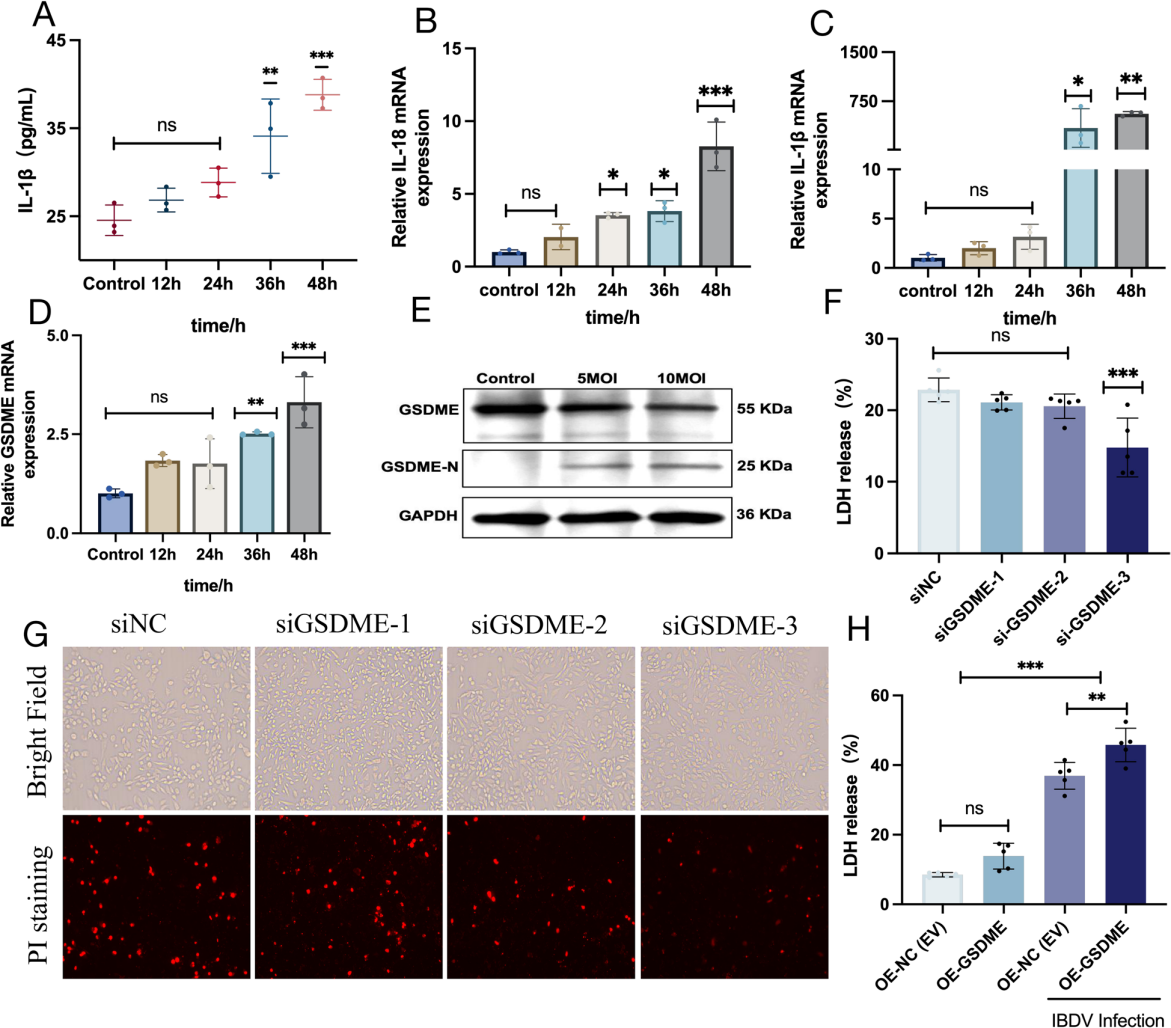
**IBDV induces inflammatory cytokine responses and activates GSDME to drive GSDME-dependent pyroptosis.** **A** IL-1β secretion measured by ELISA in mock-infected and IBDV-infected cells at 12, 24, 36, and 48 h post-infection. **B**, **C** Relative mRNA expression of IL-1β (**B**) and IL-18 (**C**) at 12, 24, 36, and 48 h post-infection. **D** Relative GSDME mRNA expression at 12, 24, 36, and 48 h post-infection. **E** Immunoblot analysis of full-length GSDME and cleaved GSDME-N in IBDV-infected cells at 24 h post-infection; GAPDH served as a loading control. **F** LDH release after siRNA-mediated GSDME knockdown (siGSDME-1/2/3) versus siNC. **G** Representative bright-field images and PI uptake after GSDME knockdown. **H** LDH release in cells transfected with empty vector (EV) or over expression (OE)-GSDME with or without IBDV infection (MOI = 10, 24 h post-infection). Data are means ± SD. ELISA and qPCR: *n* = 3 biological replicates; LDH: *n* = 6 biological replicates. Immunoblots and microscopy images are representative of three independent experiments. ns, not significant; *P < 0.05; **P < 0.01; ***P < 0.001. Statistics: two-way ANOVA with Šidák’s multiple-comparisons test (**A-D and H**) and one-way ANOVA with Dunnett’s multiple-comparisons test (**F**).

To determine whether IBDV induces GSDME-dependent pyroptosis-like lytic death, we assessed inflammatory cytokine responses and GSDME activation. ELISA showed that IL-1β secretion increased progressively following infection (Figure 2A). Consistently, the transcript levels of IL-1β and IL-18 were upregulated over time (Figure 2B, C). In parallel, GSDME mRNA expression was markedly induced after infection (Figure 2D). Immunoblotting revealed the appearance of the cleaved N-terminal fragment of GSDME (GSDME-N), accompanied by a reduction in full-length GSDME, and GSDME cleavage was enhanced with increasing viral input (Figure 2E), indicating infection-associated GSDME activation. Functionally, siRNA-mediated depletion of GSDME reduced LDH release (Figure 2F) and decreased PI-positive cells after infection (Figure 2G). Conversely, ectopic expression of GSDME potentiated IBDV-induced LDH release compared with the empty-vector control (Figure 2H). Collectively, these data indicate that IBDV induces inflammatory cytokine-associated responses and activates GSDME, thereby promoting a GSDME-dependent lytic cell death program consistent with pyroptosis.

### IBDV activates caspase-3 to cleave GSDME and promote pyroptosis-like lytic death

**Figure 3:**
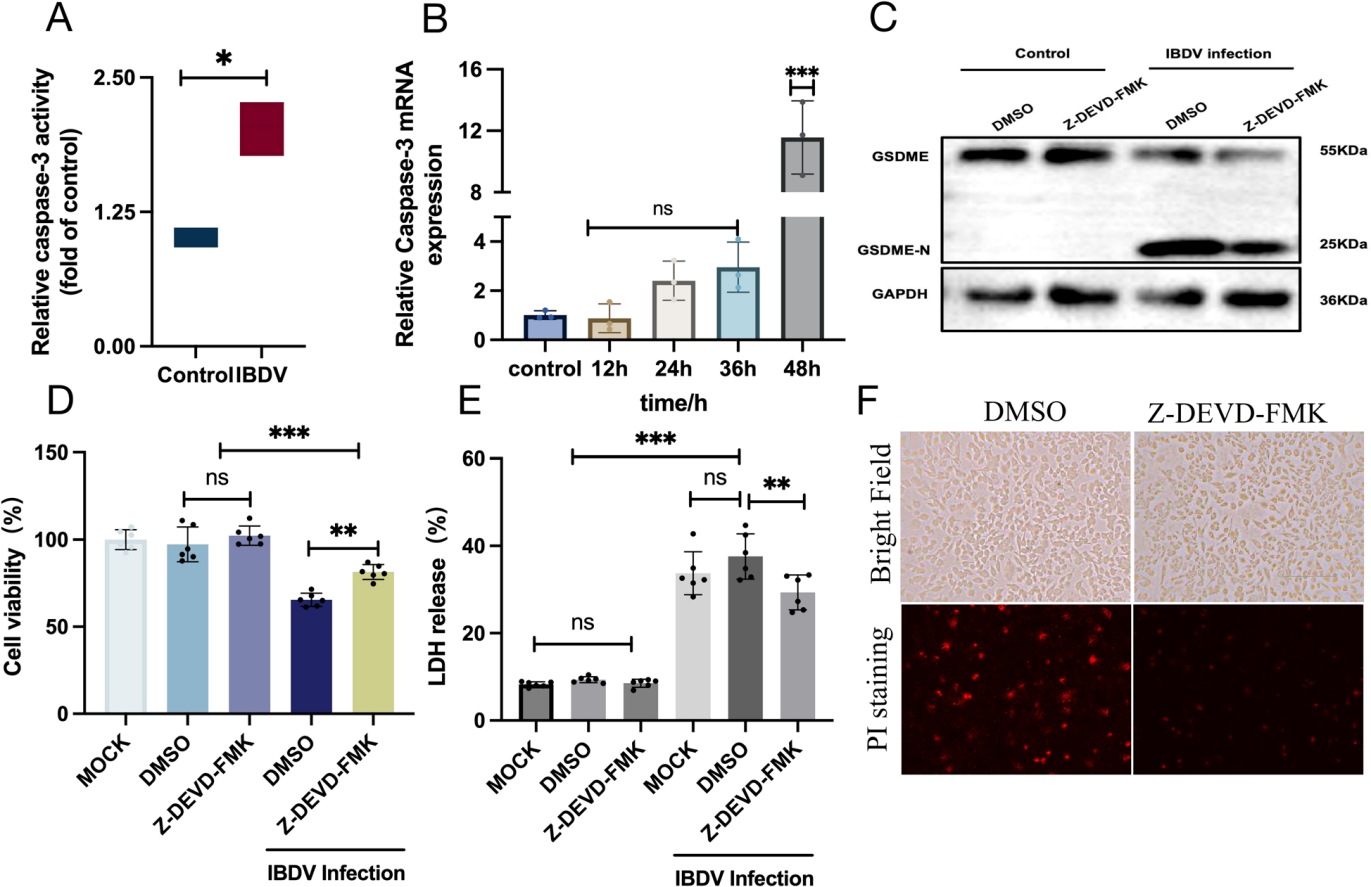
**Caspase-3 inhibition attenuates IBDV-induced GSDME cleavage and lytic cell death.** **A** Caspase-3 activity in mock-infected and IBDV-infected cells. **B** Relative caspase-3 mRNA expression in mock-infected and IBDV-infected cells at 12, 24, 36, and 48 h post-infection. **C** Immunoblot analysis of full-length GSDME and cleaved GSDME-N in DMSO- or Z-DEVD-FMK-treated cells with or without IBDV infection at 24 h post-infection; GAPDH served as a loading control. **D** CCK-8 cell viability and **E** LDH release under the indicated conditions at 24 h post-infection (MOI = 10). **F** Representative bright-field images and PI uptake under the indicated conditions at 24 h post-infection. Data are means ± SD. Caspase-3 activity and qPCR: *n* = 3 biological replicates; LDH and CCK-8: *n* = 6 biological replicates. Immunoblots and microscopy images are representative of three independent experiments. ns, not significant; *P < 0.05; **P < 0.01; ***P < 0.001. Statistics: unpaired two-tailed Student’s *t* test (**A**), one-way ANOVA with Dunnett’s multiple-comparisons test (**B**), and two-way ANOVA with Šidák’s multiple-comparisons test (**D and E**).

Given that caspase-3 is a key protease capable of cleaving GSDME, we next examined whether caspase-3 mediates GSDME processing during IBDV infection. Caspase-3 enzymatic activity was significantly increased in IBDV-infected cells compared with controls (Figure 3A), and caspase-3 mRNA expression was upregulated over the course of infection (Figure 3B). Immunoblotting revealed robust GSDME cleavage with accumulation of the N-terminal fragment (GSDME-N) after IBDV infection, whereas treatment with the caspase-3 inhibitor Z-DEVD-FMK markedly attenuated GSDME cleavage (Figure 3C). Functionally, Z-DEVD-FMK improved cell viability (Figure 3D), reduced LDH release (Figure 3E), and decreased PI-positive cells (Figure 3F), indicating alleviation of membrane damage-associated lytic death. Collectively, these data indicate that IBDV activates caspase-3 to drive GSDME cleavage and thereby contributes to infection-induced lytic cell death, which can be mitigated by pharmacological inhibition of caspase-3.

### Caspase-9 activity contributes to IBDV-induced membrane rupture and lytic cell death

**Figure 4:**
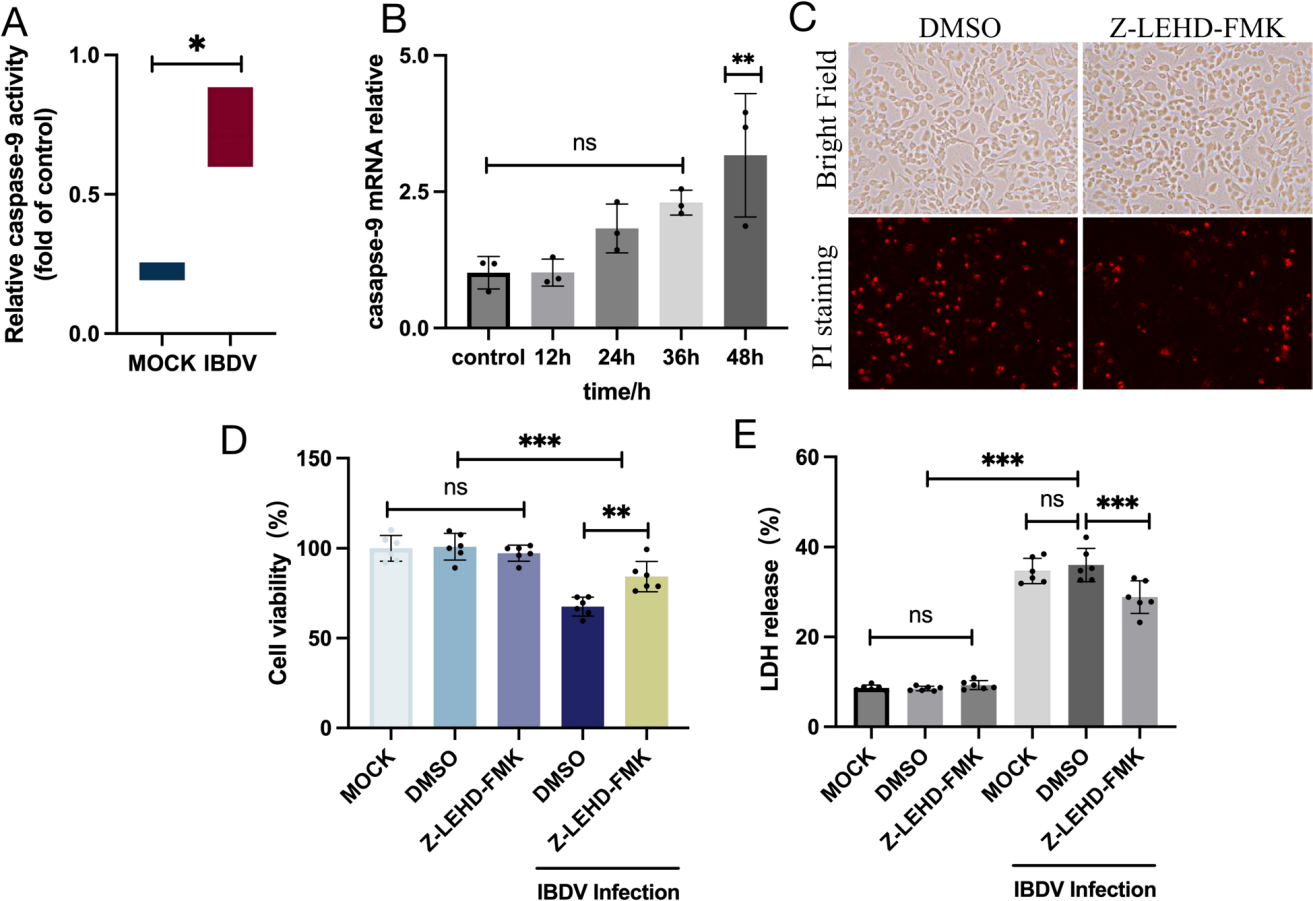
**Caspase-9 inhibition attenuates IBDV-induced membrane damage and lytic injury.** **A** Caspase-9 activity in mock-infected and IBDV-infected cells at 24 h post-infection. **B** Relative caspase-9 mRNA expression in mock-infected and IBDV-infected cells at 12, 24, 36, and 48 h post-infection. **C** Representative bright-field images and PI uptake in DMSO- or Z-LEHD-FMK-treated cells under IBDV infection at 24 h post-infection. **D** CCK-8 cell viability and **E** LDH release with or without IBDV infection at 24 h post-infection (MOI = 10). Data are means ± SD. Caspase-9 activity and qPCR: *n* = 3 biological replicates; LDH and CCK-8: *n* = 6 biological replicates. Microscopy images are representative of three independent experiments. ns, not significant; *P < 0.05; **P < 0.01; ***P < 0.001. Statistics: unpaired two-tailed Student’s *t* test (**A**), one-way ANOVA with Dunnett’s multiple-comparisons test (**B**), and two-way ANOVA with Šidák’s multiple-comparisons test (**D and E**).

Given that caspase-3 drives GSDME cleavage during IBDV infection (Figure 3), we next examined whether the upstream initiator caspase-9 is engaged. IBDV infection significantly increased caspase-9 enzymatic activity (Figure 4A) and induced a time-dependent upregulation of caspase-9 mRNA expression (Figure 4B). Pharmacological inhibition of caspase-9 with Z-LEHD-FMK reduced PI-positive cells (Figure 4C), partially restored cell viability (Figure 4D), and decreased LDH release under IBDV infection (Figure 4E), indicating attenuation of membrane damage and lytic injury. Together, these data support a role for caspase-9 in promoting IBDV-induced lytic cell death and are consistent with involvement of the caspase-9/caspase-3/GSDME axis.

### VP2 and VP5 contribute to IBDV-associated cytotoxicity and elicit caspase-inhibitor-sensitive injury

**Figure 5:**
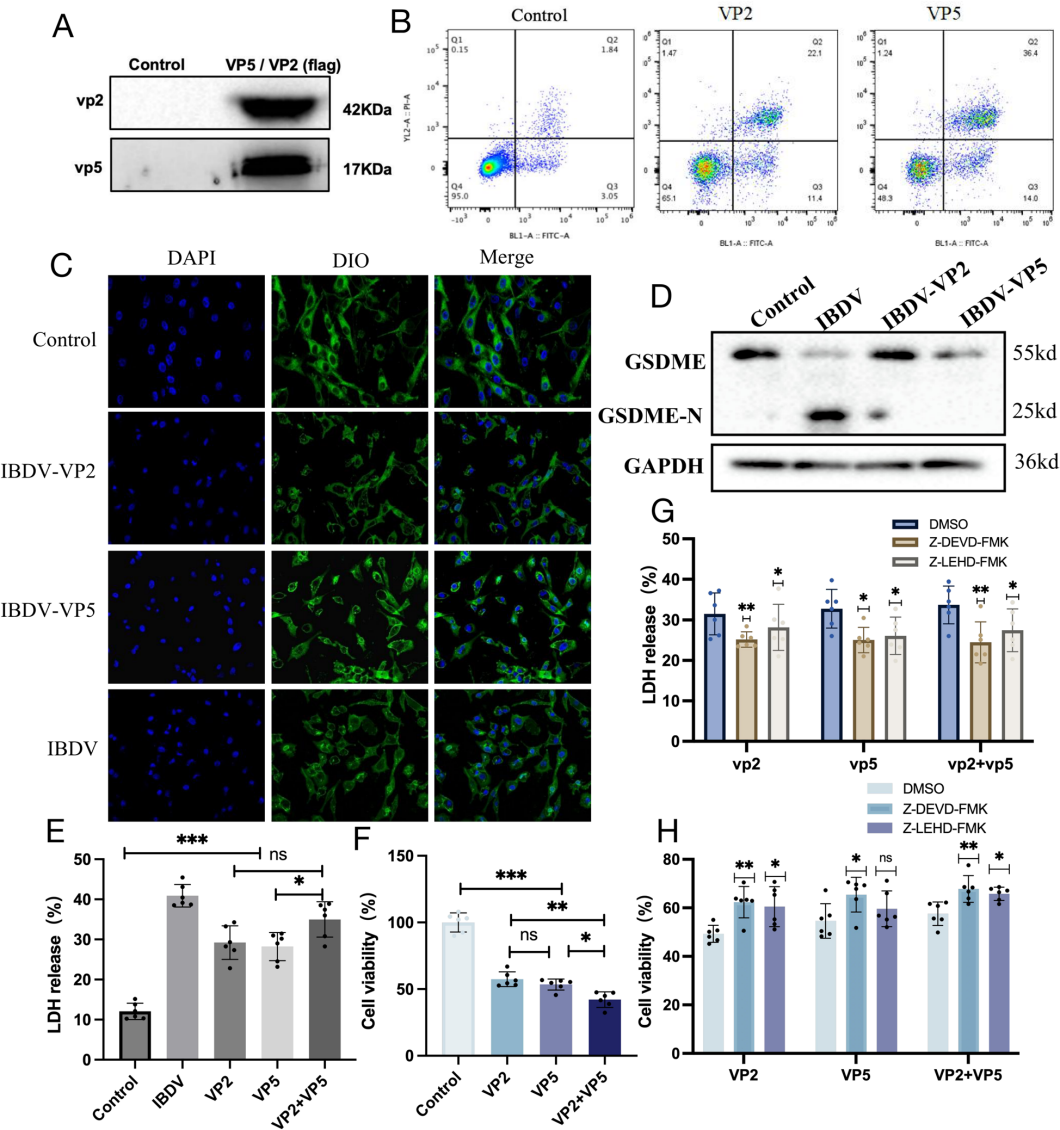
**VP2 and VP5 induce cytotoxicity and membrane damage that are partially attenuated by caspase inhibitors.** **A** Immunoblot detection of FLAG-tagged VP2 and VP5 expression in DF-1 cells after plasmid transfection. Empty vector-transfected cells served as the control. **B** Annexin V/PI flow cytometry of empty vector, VP2, and VP5 transfected cells. The empty vector group was included as the transfection control. **C** Confocal microscopy of plasma membrane staining (green) and nuclei (DAPI, blue); scale bar, 20 μm. **D** Immunoblot analysis of GSDME cleavage (GSDME-N) in control, IBDV-infected, VP2, and VP5 expressing cells. **E** LDH release and **F** CCK-8 cell viability in control, VP2, VP5, and VP2 + VP5 groups. **G** LDH release and **H** CCK-8 viability with DMSO, Z-DEVD-FMK, or Z-LEHD-FMK treatment. Unless otherwise stated, plasmid-transfected cells were analyzed at 36 h post-transfection. Data are means ± SD (*n* = 6 biological replicates). Immunoblots, flow plots, and microscopy images are representative of three independent experiments. ns, not significant; *P < 0.05; **P < 0.01; ***P < 0.001. Statistics: one-way ANOVA with Tukey’s multiple-comparisons test (**E and F**) and two-way ANOVA with Šidák’s multiple-comparisons test (**G and H**).

To determine whether individual IBDV proteins contribute to cellular injury, DF-1 cells were transfected to express VP2 and/or VP5, and protein expression was confirmed by immunoblotting using an anti-FLAG antibody (Figure 5A). Compared with the empty vector control, transfection with VP2 or VP5 increased the proportion of Annexin V/PI-positive cells (Figure 5B). Consistently, confocal imaging revealed altered plasma membrane morphology in cells expressing VP2 and/or VP5 (Figure 5C). We next assessed whether VP2/VP5 expression was associated with GSDME processing. Immunoblotting showed robust GSDME cleavage with clear generation of the GSDME-N fragment in IBDV-infected cells, whereas VP2 or VP5 overexpression alone did not produce detectable GSDME-N under the conditions tested (Figure 5D). Functionally, expression of VP2 or VP5 increased LDH release (Figure 5E) and reduced cell viability (Figure 5F), and coexpression of VP2 and VP5 further exacerbated these effects compared with either protein alone (Figure 5E, F). Notably, treatment with the caspase-3 inhibitor Z-DEVD-FMK or the caspase-9 inhibitor Z-LEHD-FMK reduced LDH release (Figure 5G) and partially restored cell viability (Figure 5H) in VP2-, VP5-, or VP2 + VP5-expressing cells. Together, these results suggest that VP2 and VP5 contribute to cytotoxicity and membrane damage, and that these effects are at least partially sensitive to caspase inhibition.

### Mitochondrial dysfunction contributes to IBDV- and VP2/VP5-associated lytic injury

**Figure 6:**
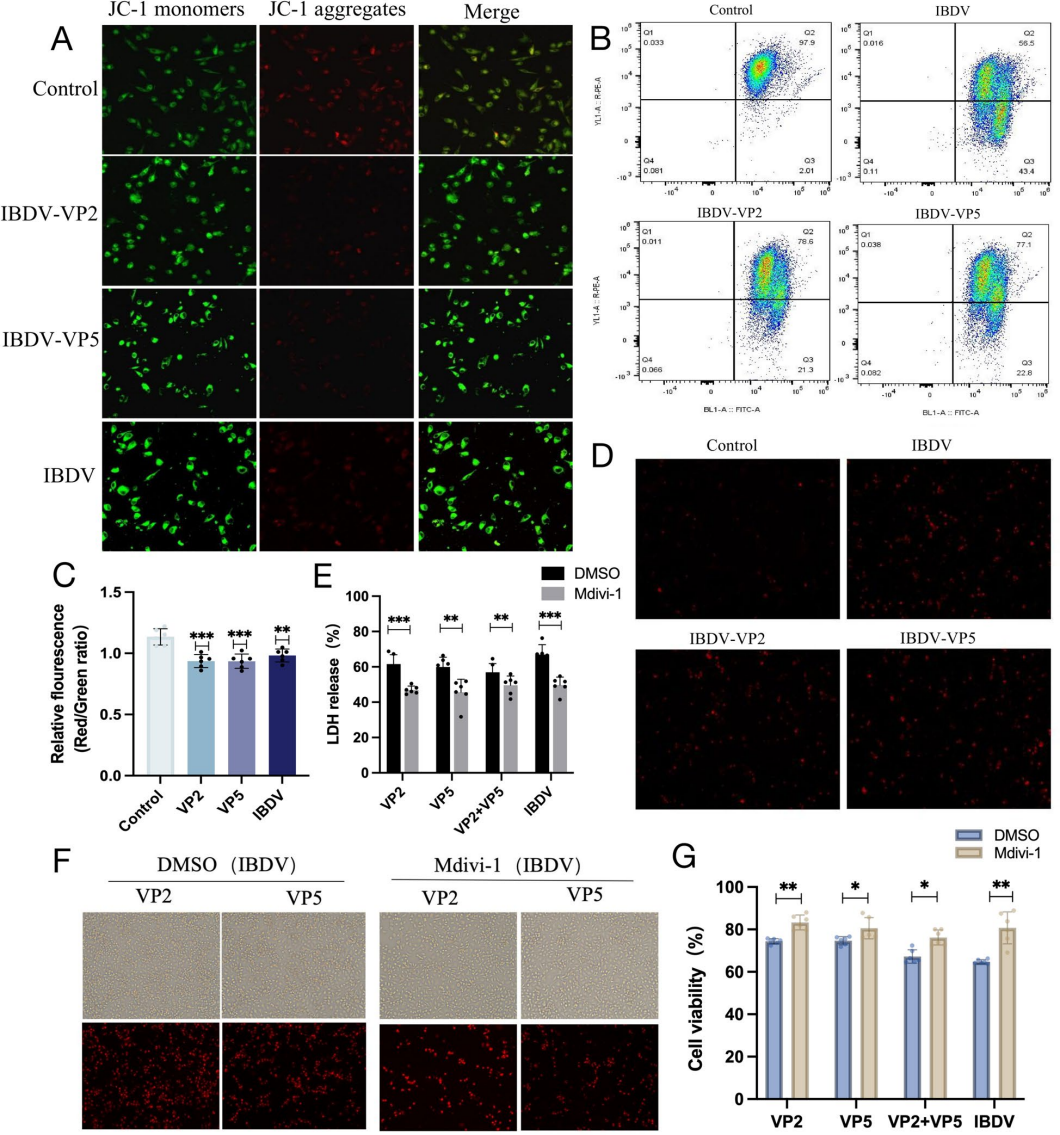
**Mitochondrial dysfunction contributes to IBDV/VP2/VP5-associated cytotoxicity.** **A** Confocal microscopy of JC-1 aggregates (red) and monomers (green) in control, VP2, VP5, and IBDV-treated cells. IBDV-infected cells were analyzed at 24 h post-infection, and plasmid-transfected cells were analyzed at 36 h post-transfection; scale bar, 20 μm. **B** Representative flow cytometry plots of JC-1 staining. **C** Quantification of the JC-1 red/green fluorescence ratio. **D** Fluorescence microscopy of intracellular ROS signals detected by MitoSOX staining. **E** LDH release, **F** representative bright-field images with PI uptake, and **G** CCK-8 cell viability in VP2, VP5, VP2 + VP5, or IBDV-treated cells in the presence of DMSO or Mdivi-1. DMSO-treated cells served as vehicle controls. Quantitative data **(C, E, and G**) are means ± SD (*n* = 6 biological replicates). Microscopy and flow cytometry plots are representative of three independent experiments. ns, not significant; *P < 0.05; **P < 0.01; ***P < 0.001. Statistics: one-way ANOVA with Tukey’s multiple-comparisons test (**C**) and two-way ANOVA with Šidák’s multiple-comparisons test **(E and G**).

To evaluate whether mitochondrial dysfunction contributes to IBDV- or viral protein-induced lytic injury, mitochondrial membrane potential was assessed using JC-1. Confocal imaging showed reduced red aggregate fluorescence and increased green monomer fluorescence in IBDV-, VP2-, and VP5-treated cells (Figure 6A), and flow cytometric JC-1 analysis further supported this shift (Figure 6B). Quantification confirmed a decreased JC-1 red/green fluorescence ratio (Figure 6C), indicating mitochondrial depolarization. In parallel, MitoSOX imaging revealed increased ROS accumulation under these conditions (Figure 6D). Functionally, the mitochondrial division inhibitor Mdivi-1 reduced LDH release (Figure 6E), decreased PI-positive cells (Figure 6F), and partially restored cell viability (Figure 6G). Together, these data suggest that mitochondrial dysfunction contributes to IBDV- and VP2/VP5-associated membrane damage and cytotoxicity.

## Discussion

IBDV infection in DF-1 cells produced a clear lytic cell-death phenotype, as shown by increased LDH release, loss of plasma membrane integrity, GSDME cleavage, and caspase-3 activation. These findings extend the current understanding of IBDV-induced cytotoxicity. Apoptosis has long been recognized as a major outcome of IBDV infection [1–3]. However, our results indicate that IBDV can also induce a GSDME-linked death program with inflammatory and lytic features. This interpretation is consistent with recent work in chicken cells showing that GSDME functions as a pore-forming effector during RNA virus infection, including IBDV-related settings [18]. Similar evidence has also been reported in ducks, where duck GSDME was identified as a caspase-3/-7 substrate and an executor of pyroptosis [20]. Nevertheless, the present study was performed in DF-1 cells. Whether this pathway directly contributes to bursal injury in vivo still needs to be examined in more relevant target cells and animal models.

The caspase network activated during IBDV infection is unlikely to represent a single linear pathway. In the classical intrinsic apoptotic pathway, mitochondrial outer membrane permeabilization promotes apoptosome formation and caspase-9 activation, which subsequently leads to caspase-3 activation [21–23]. In our study, IBDV infection increased caspase-9 activity, whereas caspase-9 inhibition reduced PI uptake, LDH release, and cell viability loss. These results place caspase-9 upstream of the lytic injury observed in this system. Previous studies further support a connection between IBDV proteins, mitochondrial stress, and caspase signaling. VP2 has been reported to promote apoptosis through ORAOV1 degradation [24], whereas VP5 can modulate mitochondrial apoptotic signaling through its interaction with VDAC2 and related host factors [13, 25]. In parallel, inflammasome-associated signaling may also participate in DF-1 cell responses to IBDV, as NLRP3-related pathways have been implicated in IBDV infection [26]. Host transcriptional responses may further shape the balance of death-related signaling during infection [9, 27]. Together, these findings suggest that IBDV may engage multiple cell-death pathways, and the dominant program may vary depending on the cell type, infection stage, and experimental context.

Mitochondrial dysfunction was closely associated with the lytic phenotype observed in our study. IBDV infection caused a decrease in mitochondrial membrane potential and an increase in ROS production, whereas Mdivi-1 partially reduced LDH release and PI uptake and restored cell viability. These findings link mitochondrial stress to the membrane-damaging outcome induced by IBDV infection. However, the Mdivi-1 data should be interpreted with caution. Although Mdivi-1 is widely used as an inhibitor of dynamin-related protein 1 (DRP1)-dependent mitochondrial fission [28, 29], it can also act as a reversible inhibitor of mitochondrial complex I and modulate ROS production [30]. Therefore, genetic approaches, such as DRP1 knockdown or rescue experiments, will be needed to determine whether mitochondrial fission itself is required for this process. Previous studies have also shown that gasdermin pores can permeabilize mitochondria and amplify caspase-3 activation [19]. Thus, our data are consistent with a model in which mitochondrial dysfunction is not merely a downstream consequence of infection, but may actively reinforce caspase activation and GSDME processing.

The viral proteins themselves also appear to contribute to this pathway. Both VP2 and VP5 induced cytotoxicity and membrane damage, and these effects were partially relieved by caspase inhibition. These results are consistent with previous reports showing that VP2 can promote apoptotic signaling and that VP5 can regulate mitochondrial apoptosis through host interactions [13, 24, 25]. However, neither VP2 nor VP5 alone fully reproduced the robust GSDME cleavage observed in IBDV-infected cells. This difference suggests that efficient GSDME activation may require additional signals generated during whole-virus infection, such as viral replication, broader mitochondrial stress, or innate immune activation. In this sense, the complete viral infection context appears to be important for full activation of the caspase-9/3-GSDME axis.

Apoptosis and pyroptosis-like death are mechanistically distinct forms of regulated cell death, but they can intersect during infection and inflammatory stress [31, 32]. Gasdermins are central executors of lytic inflammatory cell death because their N-terminal fragments form membrane pores after proteolytic activation [33–35]. In this context, GSDME provides a direct bridge between apoptotic caspase signaling and pyroptosis-like membrane rupture: caspase-3-mediated cleavage of GSDME can convert apoptotic signaling into lytic cell death [16, 17]. This concept is also supported by avian studies showing that chicken and duck GSDME can act as caspase-cleaved pore-forming effectors during virus-induced pyroptosis [18, 20]. Mitochondrial stress may further reinforce this transition, as gasdermin pores can permeabilize mitochondria and amplify caspase-3 activation [19].

**Figure 7:**
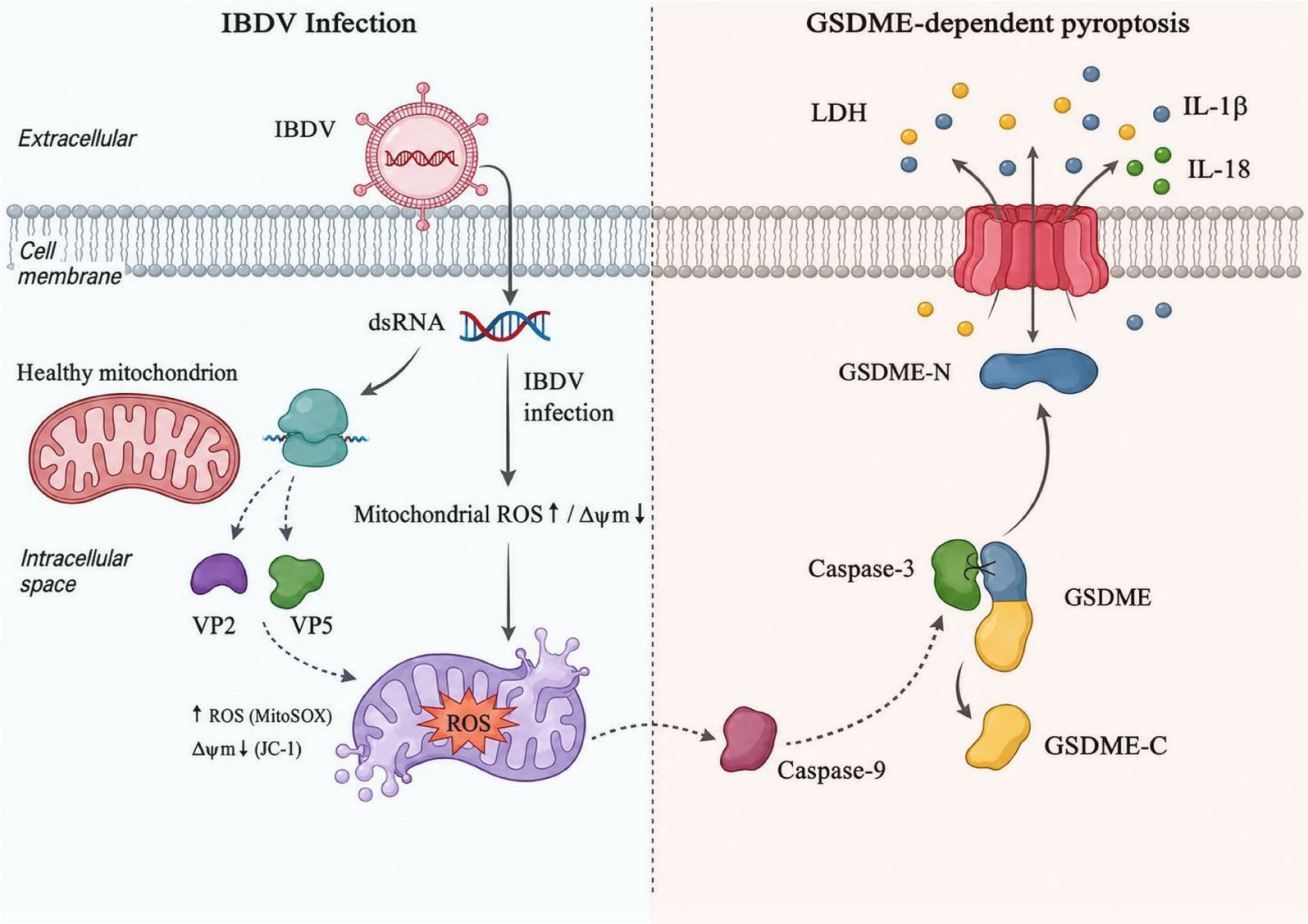
**Proposed model of IBDV-induced GSDME-dependent pyroptosis-like lytic injury in DF-1 cells.** IBDV infection is associated with mitochondrial dysfunction, including increased mitochondrial ROS and loss of mitochondrial membrane potential (Δψm). These changes coincide with activation of caspase-9 and caspase-3 and caspase-3-mediated cleavage of GSDME to generate the pore-forming GSDME-N fragment. GSDME-N is proposed to insert into the plasma membrane to form pores, resulting in membrane permeabilization, LDH release, and inflammatory cytokine responses, including IL-1β secretion and induction of IL-18 expression.

In DF-1 cells, our data support a model in which IBDV activates a mitochondria-associated caspase-9/3-GSDME axis and shifts cell death from a purely non-lytic apoptotic pattern toward a more lytic form (Figure 7). This model remains provisional. Future studies using viral mutants, rescue experiments, and genetic manipulation of mitochondrial regulators and gasdermins will be required to define which steps are directly induced by IBDV and which are secondary consequences of infection.

Overall, our results identify a mitochondria-associated caspase-9/3-GSDME axis as an important contributor to IBDV-induced lytic injury in DF-1 cells. This pathway may influence inflammatory mediator release and virus-associated tissue damage. Interfering with mitochondrial stress or caspase/GSDME signaling may therefore be worth exploring in follow-up studies, although the physiological relevance of this mechanism needs to be established in more relevant cell types and in vivo models.

## Supplementary Information

**Additional file 1.**  **Primer sequences used in this study**. Sequences of primers used for quantitative real-time PCR analysis.

**Additional file 2.**  **siRNA sequences used in this study.** Sequences of siRNAs targeting chicken GSDME (DFNA5).

## Data Availability

All data generated or analyzed during this study are included in this published article and its Additional file 1.
