# Supplementary material for: Infectious bursal disease virus induces GSDME-dependent pyroptosis via the mitochondrial caspase-9/3 pathway in DF-1 cells
Source: Vet Res. 2026 Aug 12;57:148. doi: 10.1186/s13567-026-01815-w (PMC13471472; doi:10.1186/s13567-026-01815-w)
Supplement: Supplementary file 1 [file 13567_2026_1815_MOESM1_ESM.docx]

**Additional file 1**

*Manuscript: Infectious bursal disease virus induces GSDME-dependent pyroptosis via the mitochondrial caspase-9/3 pathway in DF-1 cells*

**Title: Primer sequences used in this study**

Description: Description: Sequences of primers used for quantitative real-time PCR analysis.

| **Gene** | **Forward primer (5′→3′)** | **Reverse primer (5′→3′)** |
| --- | --- | --- |
| caspase-3 | ATCTCGTTCAGGCACGGATG | AAACTGCTTCGCTTGCTGTG |
| caspase-9 | AACCTTGGACAGCGTACTGG | CGATGTCTGACACCCGAAGT |
| GSDME | AATGAAGTGGGCCCTCAGTG | AGTAGTGGTTGGCAAAAGCAA |
| IL-18 | GCAGTACGGCTTAGAGAAAA | GTACATTCCACTGCCAGATT |
| IL-1β | TGTCAGCAAAGTCCCTGCTC | CTGCCTGCAGAAGAAGCCT |
