# Supplementary material for: Infectious bursal disease virus induces GSDME-dependent pyroptosis via the mitochondrial caspase-9/3 pathway in DF-1 cells
Source: Vet Res. 2026 Aug 12;57:148. doi: 10.1186/s13567-026-01815-w (PMC13471472; doi:10.1186/s13567-026-01815-w)
Supplement: Supplementary file 2 [file 13567_2026_1815_MOESM2_ESM.docx]

**Additional file 2**

*Manuscript: Infectious bursal disease virus induces GSDME-dependent pyroptosis via the mitochondrial caspase-9/3 pathway in DF-1 cells*

**Title: siRNA sequences used in this study**

Description: Sequences of siRNAs targeting chicken GSDME (DFNA5).

| **siRNA** | **Sense (5′→3′)** | **Antisense (5′→3′)** |
| --- | --- | --- |
| siGSDME-1 | UUUACUAUCUUCGUAGUGCTT | GCACUACGAAGAUAGUAAATT |
| siGSDME-2 | GGACAGAAUGAUAAAUUUATT | UAAAUUUAUCAUUCUGUCCTT |
| siGSDME-3 | AGCUAUUGUUGCAGAACUATT | UAGUUCUGCAACAAUAGCUTT |
